# Independent and incremental prognostic value of D-dimer in hospitalized COVID-19 patients

**DOI:** 10.2217/fvl-2021-0161

**Published:** 2021-07-29

**Authors:** Gerald Chi, Sahar Memar Montazerin, Jane J Lee

**Affiliations:** 1 ^1^Division of Cardiovascular Medicine, Beth Israel Deaconess Medical Center, Harvard Medical School, Boston, MA 02215, USA; 2 ^2^Department of Trial Design and Development, Baim Institute for Clinical Research, Boston, MA 02215, USA

**Keywords:** biomarkers, coronavirus, COVID-19, D-dimer, venous thrombosis

The outbreak of COVID-19 has affected billions of people worldwide. The clinical spectrum of COVID-19 ranges from asymptomatic infection or mild disease to severe pneumonia manifesting as respiratory failure, septic shock or multiple organ dysfunction. An early cohort study demonstrated that approximately 19% of symptomatic patients with COVID-19 had severe diseases or became critically ill [1]. Aside from respiratory symptoms, severe COVID-19 is associated with a substantial risk of thromboembolic complications, with an estimated incidence of approximately 10–20% among hospitalized patients despite the use of pharmacological thromboprophylaxis based on meta-analyses of observational studies [2,3]. The thromboembolic complications have been explained by the interplay between the coronavirus and endothelial cells, local and systemic inflammatory response and coagulation system [4].

D-dimer is a specific degradation product of crosslinked fibrin clots and has been routinely used in clinical practice to aid the diagnosis of venous thromboembolism (VTE). Apart from its diagnostic value, D-dimer has been linked to adverse prognosis (including mortality, incident VTE and recurrent VTE) in various clinical settings [5]. In randomized controlled trials of acutely-ill hospitalized patients, the elevation of D-dimer >two-times the upper limit of normal (ULN) was independently associated with a twofold to threefold greater risk of VTE within 90 days of hospital admission, indicating that D-dimer can be used to identify high-risk subsets of medical inpatients [6,7]. Further analysis demonstrated that patients with D-dimer >2 × ULN had a greater VTE reduction and comparable bleeding risk than those with D-dimer ≤2 × ULN, which supports its use in assisting decision making on extended VTE prophylaxis for acutely-ill medical patients [8]. With respect to the incremental prognostic value, D-dimer was shown to enhance the performance of the VTE risk-assessment model when incorporated into the International Medical Prevention Registry on Venous Thromboembolism (IMPROVE) score, by refining its capacity of discriminating and reclassifying VTE events [9]. A retrospective analysis confirmed that the combination of IMPROVE score with D-dimer measurement identified a high-risk subpopulation of acutely-ill medical patients where a significant benefit of extended or post-discharge VTE prophylaxis could be derived [10].

In light of the heightened risk of VTE in severe COVID-19 patients, a question concerning D-dimer arises as to whether the independent and incremental prognostic value of this biomarker can be extrapolated from non-COVID-19 populations to COVID-19 populations. Meta-analyses have shown that among hospitalized COVID-19 patients, those who developed VTE had a significantly higher D-dimer at baseline than those who did not develop VTE, with a mean difference of 2–3 μg/ml [2,3]. It should be noted that there has been substantial heterogeneity across the studies regarding the association of D-dimer with VTE in COVID-19. A possible explanation is that, there may be a heterogeneous predisposition to thrombotic complications among COVID-19 patients. Alternatively, the heterogeneity may reflect the difference in the methodology employed to measure D-dimer levels or identify VTE events. In regards to the incremental prognostic information of D-dimer, a single-center observational study revealed that the combined use of D-dimer >1.0 μg/ml, CURB-65 score (i.e., confusion status, urea, respiratory rate and blood pressure) 3–5, and Padua prediction score ≥4 yielded a sensitivity of 88.5% and specificity of 61.4% for screening of deep vein thrombosis [11]. In addition, the performance of IMPROVE-DD score (i.e., incorporating D-dimer into the IMPROVE VTE risk-assessment model) was investigated in a retrospective cohort study of adult patients hospitalized with COVID-19 [12]. The IMPROVE-DD score demonstrated good VTE discriminatory capacity and stratified COVID-19 patients into low, moderate and high VTE risk. Specifically, there was a stepwise increase in VTE risk with the IMPROVE-DD score category, with 0.41% for the score 0–1 (low risk), 1.21% for score 2–3 (moderate risk) and 5.30% for the score ≥4 (high risk). These findings support the implementation of D-dimer measurement in conjunction with a validated VTE risk-assessment model for stratifying COVID-19 patients requiring hospital admission.

Despite the demonstrated prognostic value of D-dimer in the context of COVID-19, several issues regarding the utilization of D-dimer have been recently addressed by the Scientific and Standardization Committee (SSC) on Fibrinolysis of the International Society on Thrombosis and Haemostasis (ISTH) [13]. The SSC pointed out that the type of D-dimer assay and its analytical performance have not been consistently reported in the majority of the COVID-19 studies. Additionally, the cutoff value of D-dimer varies widely in the COVID-19 literature. For instance, in the study by Zhou and colleagues, D-dimer >1.0 μg/ml was associated with 18-fold increased odds for in-hospital death, while the association was not significant using the cutoff of >0.5 μg/ml [14]. Similarly, in the study by Tang and colleagues, the survival benefit associated with heparin was evident in patients with D-dimer >6 × ULN but not in the overall population [15]. As D-dimer level is frequently elevated in COVID-19 patients and the conventional D-dimer cutoff of 0.5 μg/ml may have inadequate specificity for predicting adverse outcomes or guiding thromboprophylaxis decisions in COVID-19 patients. However, there is no clear consensus on the optimal D-dimer cutoff for risk-stratifying COVID-19 patients. The SSC indicated that, rather than standardization of D-dimer assays, harmonization of D-dimer test results by converting D-dimer values from different assays to a common scale may be a possible solution to these issues [16].

The ISTH interim guidance recommends checking D-dimer, prothrombin time and platelet count (in decreasing order of importance) in all patients who present with COVID-19 infection [17]. In this scenario, a threefold to fourfold increase in D-dimer values may be considered markedly elevated and hospital admission with close monitoring may be warranted. In addition, the ISTH interim guidance suggests serial monitoring of these parameters to assist in determining the prognosis in hospitalized COVID-19 patients. Stabilization of clinical condition and coagulation parameters may add confidence to stepdown of treatment, while clinical deterioration with marked elevation of D-dimer may warrant diagnostic workup for potential thrombotic complications or more aggressive critical care support. At last, it should be stressed that there has been insufficient evidence to support the use of D-dimer as a standalone tool in clinical decision making on the escalation of anticoagulation therapy, as this strategy has not been tested in randomized controlled trials [18].

## Future perspective

VTE risk stratification in hospitalized COVID-19 patients remains an ongoing challenge and imprecise science. Among the biomarkers that have been demonstrated to be predictive of adverse prognosis, D-dimer appears to be one of the most promising laboratory tests because of its biological plausibility and supportive evidence. First, as a hallmark of COVID-19-associated coagulopathy, markedly elevated D-dimer levels reflect the dysregulated fibrinolysis in the local alveoli by urokinase-type plasminogen activator released from alveolar macrophages. Second, numerous clinical studies have consistently demonstrated that D-dimer was independently associated with a greater risk of thromboembolic complications, which were the main contributors to morbidity and mortality in COVID-19 patients. Third, D-dimer offers incremental prognostic information to VTE risk assessment model such as the IMPROVE score. Specifically, the addition of D-dimer to the IMPROVE score significantly enhanced the VTE discriminatory performance by 7%, as assessed by the area under the receiver-operating characteristic curve [12]. Consequently, the incorporation of D-dimer into VTE assessment strategies for COVID-19 patients has been recommended in real-world clinical settings [19,20]. Taken together, given the independent and incremental prognostic utility of D-dimer, future research should determine whether the conjunctive use of D-dimer measurement and VTE risk-assessment model impacts the practice of VTE prophylaxis and the rate of thromboembolic complications in COVID-19 patients.
